# HOW OXIDATIVE DAMAGE TO TELOMERES PROMOTES PREMATURE CELLULAR SENESCENCE

**DOI:** 10.1093/geroni/igae098.1143

**Published:** 2024-12-31

**Authors:** Patricia Opresko

**Affiliations:** University of Pittsburgh, Pittsburgh, Pennsylvania, United States

## Abstract

Telomeres are protective caps at chromosome ends required for genome stability and sustained cell proliferation. Oxidative stress is associated with accelerated telomere shortening and dysfunction, and telomeric sequences are highly susceptible to oxidative damage. To study how oxidative damage impacts telomere function, we use a chemoptogenetic tool that produces the common oxidative lesion 8-oxoguanine (8oxoG) selectively at telomeres. Human cancer cells =are largely unaffected by a single induction of telomeric 8oxoG, but chronic damage impairs cell growth and increases telomere shortening and loss. In contrast, a single induction of telomeric 8oxoG in non-disease human fibroblast and epithelial cells triggers hallmarks of p53-dependent cellular senescence. Telomeric 8oxoG promotes replication stress at telomeres, leading to telomere fragility rather than accelerated telomere shortening. Surprisingly, the loss of repair enzymes OGG1 and MUTYH partially rescues the telomeric 8oxoG-induced senescence and telomere dysfunction, while double knockout causes a near complete rescue. These glycosylases initiate base excision repair at sites of 8oxoG, leading to repair intermediates, including abasic sites and single strand breaks. Following telomeric 8oxoG damage, repair intermediates accumulate at telomeres in wild type, but not OGG1/MUTYH deficient cells. These data suggest that inefficient completion of 8oxoG BER at telomeres triggers cellular senescence via repair intermediates which disrupt telomere function. We uncovered a novel mechanism for telomere-driven rapid senescence that depends on DNA replication and glycosylase activity but occurs in the absence of telomere shortening. This mechanism may contribute to markers of telomere dysfunction that increase with age and oxidative stress in slowly proliferating tissues.

